# Comparative Bioaccessibility of Phenolic Compounds from Almonds, Peanuts, Pistachios and Their Corresponding Butters

**DOI:** 10.3390/foods15132302

**Published:** 2026-06-27

**Authors:** Maddalena De Angeli, Melissa Zannini, Angela Conte, Davide Tagliazucchi

**Affiliations:** Nutritional Biochemistry Lab, Department of Life Sciences, University of Modena and Reggio Emilia, Via Amendola 2, 42122 Reggio Emilia, Italy; maddalena.deangeli@unimore.it (M.D.A.); melissa.zannini@unimore.it (M.Z.); davide.tagliazucchi@unimore.it (D.T.)

**Keywords:** mass spectrometry, polyphenols, food matrix, in vitro digestion, functional foods

## Abstract

Nuts are an important dietary source of phenolic compounds, which are associated with potential health benefits. In the present study, the bioaccessibility of phenolic compounds from almonds, peanuts, pistachios and their corresponding butters was investigated after in vitro gastro-intestinal digestion. High-resolution mass spectrometry analysis provided a comprehensive picture of the phenolic profiles across samples, with a total of 55–95 compounds identified per matrix. Pistachios showed the highest amount of phenolic compounds (60.603 ± 1.170 mg/100 g), followed by peanuts and almonds. Processing into butter affected phenolic concentration in a matrix-specific manner. Almond and peanut butters showed higher phenolic content compared to whole nuts, whereas pistachio butter exhibited lower levels than the whole nut. After digestion, differences in bioaccessibility were observed. Peanuts showed the highest phenolic bioaccessibility (84.31%), while almonds and pistachios exhibited lower values (<15%). Overall, phenolic bioaccessibility was mainly influenced by nut type and phenolic compound structure, rather than by their initial concentration or processing. These findings are based on an in vitro model representing an estimate of bioaccessibility rather than in vivo absorption. However, nut type and processing appear to influence the release of phenolic compounds, which is relevant for the nutritional evaluation of nuts and nut-derived products.

## 1. Introduction

Nuts are a key component of healthy eating patterns, including the Mediterranean diet, with a large consumption of almonds, Brazil nuts, cashews, hazelnuts, macadamia nuts, pecans, pine nuts, pistachios and walnuts. A daily intake of approximately 30–40 g of nuts is generally associated with a reduced risk of several chronic diseases [1]. The beneficial health effects of both tree nuts and peanuts are largely attributed to their rich nutritional profile, which includes vitamins, minerals, monounsaturated and polyunsaturated fatty acids, and dietary fiber. In addition, nuts contain bioactive compounds such as carotenoids, phenolic compounds, and phytosterols [1,2].

Specifically, nut consumption has been associated with a reduced risk of developing cardiovascular diseases and diabetes, as well as improvements in the lipid profile, inflammation markers, and preservation of endothelial function [3]. Nuts have also been shown to protect neurons from free radicals and other reactive oxygen species, thereby contributing to the delay of cognitive decline [4]. Their beneficial effect on brain health may also be mediated through the gut–brain axis, a bidirectional communication system between the intestine and the central nervous system that regulates behavior and contributes to the maintenance of immune homeostasis in the brain [5].

Phenolic compounds represent a heterogeneous group of natural substances produced by the secondary metabolism of plants, contributing to their defense against external pathogens. In recent years, clinical studies have demonstrated that these compounds exerted beneficial effects on human health. In particular, they have attracted considerable attention due to evidence from both in vitro and in vivo studies highlighting their antioxidant, anti-inflammatory, anti-diabetic, and anti-proliferative activities [6,7,8]. Many of the health benefits associated with vegetable food consumption have been attributed to the presence of phenolic compounds, which is why their dietary intake is highly recommended [8].

Nevertheless, one of the main factors limiting the beneficial effects of phenolic compounds is their bioaccessibility and subsequent absorption in the gastro-intestinal tract, along with their biotransformation by gut microbiota enzymes [9]. To exert their biological activity, phenolic compounds must be released from the food matrix during digestion and remain stable within the gastro-intestinal environment [10]. The release and stability of phenolic compounds in the gastro-intestinal tract are encompassed in the concept of bioaccessibility, defined as the proportion of a nutrient or phytochemical that is released from the food matrix during digestion and becomes potentially available for absorption in the gastro-intestinal tract [11].

The bioaccessibility of phenolic compounds is mainly influenced by the food matrix, their chemical structure, the physico-chemical conditions in the gastro-intestinal tract (i.e., presence of proteins, bile salts, and the pH), and the activity of digestive enzymes [12].

In addition, increasing evidence highlights that the physical state of the matrix plays a crucial role in the release, mass transfer, accessibility, and biochemical stability of many food components, due to their interaction with macromolecules, such as proteins, fibers, and lipids [13,14]. When lipids interact with phenolic compounds, they may bind and retain them, shielding them during gastro-intestinal transit and thereby facilitating their delivery. A protective role of the lipid matrix on procyanidins and flavones during duodenal digestion has been proposed, likely related to enhanced micelle formation, which improves the stability of phenolic compounds throughout digestion [13]. Otherwise, proteins may reduce the bioaccessibility of phenolic compounds through the formation of phenolic–protein complexes, which lead to the formation of insoluble aggregates [14].

Furthermore, previous studies suggested that several phenolic compounds, particularly those containing a catechol moiety, may undergo oxidative degradation under the alkaline intestinal conditions [15,16]. Moreover, other reactions like deglycosylation, hydrolysis, demethylation, and deacetylation may occur during the in vitro digestion of phenolic compounds [17].

Among the different phenolic compound classes, phenolic acids are the compounds most commonly identified in peanuts, both in the butter and in the whole nut, whereas flavonols are predominant in almonds [18]. In pistachios, however, high concentrations of phenolic compounds were not detected after in vitro digestion, likely due to their instability under gastro-intestinal conditions [19].

Despite the nutritional and health relevance of nuts, previous studies have mainly focused on identifying key phenolic compounds, rather than providing a comprehensive characterization of their phenolic profiles, particularly in comparison with their corresponding butters. In addition, information on their bioaccessibility during digestion remains limited and fragmented.

Therefore, the aim of this study was to provide a more comprehensive overview of the phenolic profiles of selected nuts and to evaluate the potential influence of the food matrix on their phenolic composition and behavior. Three commonly consumed nuts, almonds, peanuts and pistachios, and their corresponding butters were selected for analysis. Ultra-high-pressure liquid chromatography coupled with high-resolution mass spectrometry was used to determine the phenolic profile of the samples and to assess their behavior following in vitro digestion, with particular emphasis on their bioaccessibility.

## 2. Materials and Methods

### 2.1. Chemicals, Reagents and Food Samples Preparation

Pepsin, pancreatin and all the reagents utilized for in vitro gastro-intestinal digestion were obtained from Sigma-Aldrich (Milan, Italy). Chemicals for the extraction procedure and for mass spectrometry analysis were purchased from Bio-Rad (Hercules, CA, USA). Peanuts, almonds, pistachios and their corresponding butters were purchased at a local supermarket (Reggio Emilia, Italy). All samples included skin. All nut butters used in the study were prepared from 100% nuts, including the skin, without the addition of any additives.

### 2.2. Extraction of Phenolic Compounds

The extraction of phenolic compounds from nuts and their corresponding butters was carried out according to a previously established protocol [20]. Samples (1.5 g) were treated with 5 mL of a methanol/water/formic acid solution (70:28:2, *v*/*v*/*v*) and homogenized at 6000 rpm using an Ultra-Turrax device. The mixtures were subsequently stirred at 37 °C for 1 h to facilitate phenolic extraction. Following centrifugation at 6000× *g* for 10 min at 4 °C, the supernatants were collected and preserved at −80 °C until their characterization by high-resolution mass spectrometry and spectrophotometric analyses. To ensure exhaustive recovery of phenolic compounds, the extraction procedure was performed at least twice for each sample. Extraction efficiency was assessed by determining the total phenolic content using the Folin–Ciocalteu assay, as described in Section 2.4.1. The extraction procedure was performed in triplicate for each sample.

### 2.3. In Vitro Gastro-Intestinal Digestion

In vitro gastro-intestinal digestion was performed exactly as reported in the INFOGEST 2.0 protocol [21]. Food samples (1 g) were mixed with 1 mL of salivary fluid, homogenized using a pestle and mortar to mimic mastication, and then incubated at 37 °C for 2 min in a rotating wheel (10 rpm). Gastric digestion was initiated by adding 2 mL of simulated gastric fluid to the bolus, adjusting the pH to 3.0 with 6 mol/L HCl, and adding pepsin to a final activity of 2000 U/mL. Samples were then incubated for 120 min at 37 °C in a rotating wheel (10 rpm). Following the gastric phase, intestinal digestion was carried out by adding 4 mL of simulated intestinal fluid, adjusting the pH to 7.5 with concentrated NaOH, and adding pancreatin to a final activity of 200 U/mL (based on trypsin activity). Following digestion, the chyme was incubated at 37 °C for 120 min on a rotating wheel operating at 10 rpm. After completion of the digestion, samples were kept at −80 °C pending analysis of phenolic compounds by high-resolution mass spectrometry and spectrophotometric methods. All samples were digested in triplicate.

### 2.4. Determination of Total Phenolic Content and Antioxidant Activity

#### 2.4.1. Assessment of Total Phenolic Content

The total phenolic content of nut samples and their corresponding butters, including both extracts and digested fractions, was determined using the Folin–Ciocalteu method according to a previously published procedure [22]. Briefly, 25 µL of appropriately diluted sample was mixed with 1975 µL of distilled water and 125 µL of Folin–Ciocalteu reagent. After exactly 1 min of reaction, 375 µL of Na_2_CO_3_ was added to stop the reaction and allowed for color development. After 2 h, the absorbance was read at 760 nm. The data were reported as mg of gallic acid equivalent per 100 g of sample.

#### 2.4.2. ABTS Radical Scavenging Activity Assay

The antioxidant capacity of nut samples and their corresponding butters, including both extracts and digested samples, was evaluated using the ABTS radical cation decolorization assay according to the method described by Re et al. [23]. The ABTS radical cation was generated by mixing a 14 mmol/L ABTS solution with a 4.9 mmol/L potassium persulfate solution at a 1:1 (*v*/*v*) ratio and allowing the mixture to react overnight in the dark. Prior to analysis, the resulting ABTS radical solution was diluted with phosphate buffer (0.1 mol/L, pH 7.5) to achieve an absorbance of 0.8–0.9 at 734 nm. For the assay, 40 µL of suitably diluted sample was added to 1960 µL of the diluted ABTS radical solution. After incubation at room temperature for 10 min, the decrease in absorbance was measured at 734 nm. Results were expressed as mg of Trolox equivalent per 100 g of sample.

#### 2.4.3. Measurement of Ferric Reducing Antioxidant Power (FRAP)

Ferric reducing antioxidant power (FRAP) of nut samples and their corresponding butters, both before and after digestion, was determined following the method of Benzie and Strain [24]. The working FRAP reagent (WFR) was prepared by combining acetate buffer (300 mmol/L, pH 3.6), 20 mmol/L FeCl_3_, and 10 mmol/L TPTZ solution (dissolved in 40 mmol/L HCl) in a 10:1:1 (*v*/*v*/*v*) ratio. For the assay, 100 µL of suitably diluted sample was mixed with 3 mL of WFR and incubated for 7 min at room temperature. The absorbance was then measured at 593 nm. FRAP results were expressed as mg of FeSO_4_ equivalent per 100 g of sample.

### 2.5. Identification and Quantification of Phenolic Compounds by High-Resolution Mass Spectrometry

High-resolution mass spectrometry analysis was performed using a Q Exactive Hybrid Quadrupole-Orbitrap mass spectrometer coupled to a UHPLC Ultimate 3000 module (Thermo Fisher Scientific, San Jose, CA, USA). Phenolic compounds were separated using a C18 column (Acquity UPLC HSS C18 Reversed phase, 2.1 mm × 100 mm, 1.8 µm particle size, Waters, Milan, Italy) with a binary gradient consisting of water (containing 0.1% of formic acid) and acetonitrile (containing 0.1% of formic acid). The gradient program, flow rate, and all other chromatographic and mass spectrometry conditions were described by Martini et al. [25]. Data were reported as mg/100 g of sample. The bioaccessibility index (BI) was determined following the procedure described by Cattivelli et al. [26].

Information on analytical standards and detailed mass spectrometric parameters is indicated in Supplementary Table S1.

Protocatechuic acid, vanillic acid, gallic acid, 3-hydroxycinnamic acid, 4-hydroxycinnamic acid, caffeic acid, ferulic acid, epicatechin, catechin, epicatechin-3-O-gallate, catechin-3-O-gallate, epigallocatechin-3-O-gallate, procyanidin B1, procyanidin B2, apigenin-7-O-glucoside, luteolin-7-O-glucoside, naringenin, kaempferol, quercetin, quercetin-3-O-glucoside, quercetin-3-O-galactoside, kaempferol-3-O-rutinoside and quercetin-3-O-rutinoside have been identified by comparison of the retention tome and fragmentation behavior with authentic standards. All other phenolic compounds were identified based on their m/z values and fragmentation patterns reported in the literature, as well as established fragmentation rules.

### 2.6. Statistics

Quantitative data from mass spectrometric analysis of phenolic compounds and spectrophotometric assays are expressed as mean ± standard deviation. Normality and homogeneity of variance were assessed using the Shapiro–Wilk and Levene tests, respectively. Statistical comparisons were conducted using one-way ANOVA followed by Tukey’s post hoc test in GraphPad Prism 10 (GraphPad Software, San Diego, CA, USA). Differences were deemed statistically significant at *p* < 0.05. Pearson correlation analysis was performed by using GraphPad Prism 10.

## 3. Results

### 3.1. Phenolic Compound Profile and Bioaccessibility of Almonds and Almond Butter

**Figure 1:**
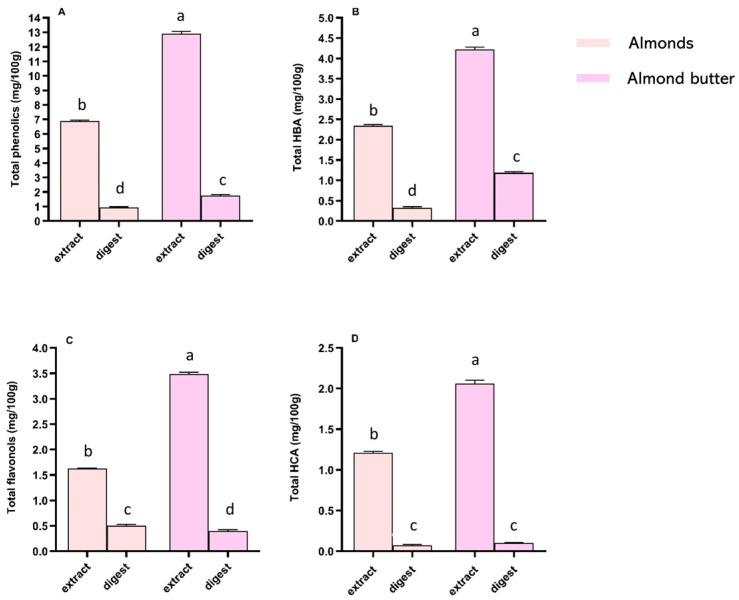
Effect of in vitro gastro-intestinal digestion on changes in total phenolic compounds (**A**), total hydroxybenzoic acids (**B**), total flavonols (**C**), and total hydroxycinnamic acids (**D**) in almonds and almond butter. “Extract” refers to phenolic compound extracts from almonds and almond butter, whereas “digest” refers to gastro-intestinally digested almonds and almond butter. Light pink bars indicate almond samples, while lilac bars indicate almond butter samples. HBA: hydroxybenzoic acids; HCA: hydroxycinnamic acids. Different letters indicate statistically significant differences (*p* < 0.05).

A total of 55 phenolic compounds were identified and quantified in almond nuts and almond butter following chemical extraction (Table 1). The total phenolic content, determined by mass spectrometry, was 6.888 ± 0.073 mg/100 g in almond nuts. A significantly higher (*p* < 0.05) concentration, 1.87-fold greater, was observed in almond butter, reaching 12.901 ± 0.160 mg/100 g (Table 1 and Figure 1A). Despite differences in total phenolic content, the relative distribution of phenolic classes was very similar between almond nuts and almond butter. Hydroxybenzoic acids were the predominant class in both samples, accounting for 34.46% and 32.74% of total phenolic compounds in almonds and almond butter, respectively (Table 1 and Figure 1B). Flavonols were the second most abundant class, representing 23.64% and 27.04% of total phenolics in almonds and almond butter, respectively, followed by hydroxycinnamic acids (17.55% and 15.99%), flavan-3-ols (17.38% and 13.02%), and flavanones (6.96% and 11.23%) (Table 1 and Figure 1C,D).

The hydroxybenzoic acid profile was dominated by di-hexoside-derivatives and their corresponding aglycones. Notably, hydroxy-methoxybenzoic acid-O-hexoside-hexoside isomer 1 was the most abundant compound identified, accounting for 18.02% and 17.77% of total phenolic compounds in almonds and almond butter, respectively (Table 1).

Among flavonols, isorhamnetin-3-O-rutinoside was the predominant compound and ranked as the second most abundant phenolic overall, representing 13.40% and 12.53% of total phenolic compounds in almond and almond butter, respectively (Table 1).

The hydroxycinnamic acid profile was also characterized by a predominance of di-hexoside-derivatives, with ferulic acid-O-hexoside-hexoside isomer 1 as the main representative (12.91% and 9.81% of total phenolic compounds in almonds and almond butter, respectively) (Table 1).

Among flavan-3-ols, (epi)catechin-O-hexoside-hexoside and procyanidin B1 were the major compounds in both matrices, whereas the monomeric forms (epicatechin and catechin) were more abundant in almond butter. Flavanones were present in comparatively low amounts, particularly in almonds (Table 1).

The bioaccessibility index (BI) of total phenolic compounds was low and comparable between samples, with values of 13.42% and 13.56% for almonds and almond butter, respectively. However, at the end of gastro-intestinal digestion, the absolute amount of bioaccessible phenolic compounds was significantly higher (*p* < 0.05) in almond butter (1.750 ± 0.068 mg/100 g) than in almonds (0.924 ± 0.057 mg/100 g) (Table 1 and Figure 1A). In almonds, flavonols exhibited the highest BI (30.96%), accounting for 54.53% of total phenolic compounds after digestion. Although isorhamnetin-3-O-rutinoside was the predominant flavonol, kaempferol-3-O-rutinoside showed the highest BI following almonds digestion (44.98% vs. 34.88%). In contrast, the BI of flavonols in almond butter was significantly lower (*p* < 0.05) (Table 1 and Figure 1C). In almond butter, hydroxybenzoic acids exhibited the highest BI (28.10%), representing 67.83% of total phenolic compounds after digestion (Table 1 and Figure 1B). Notably, vanillic acid exhibited a BI above 100% in both almonds and almond butter (Table 1). Finally, hydroxycinnamic acids and flavanones exhibited very low BI values in both samples, and flavan-3-ols showed particularly limited bioaccessibility, with BI values below 1% (Table 1 and Figure 1D).

### 3.2. Phenolic Compound Profile and Bioaccessibility of Peanuts and Peanut Butter

**Figure 2:**
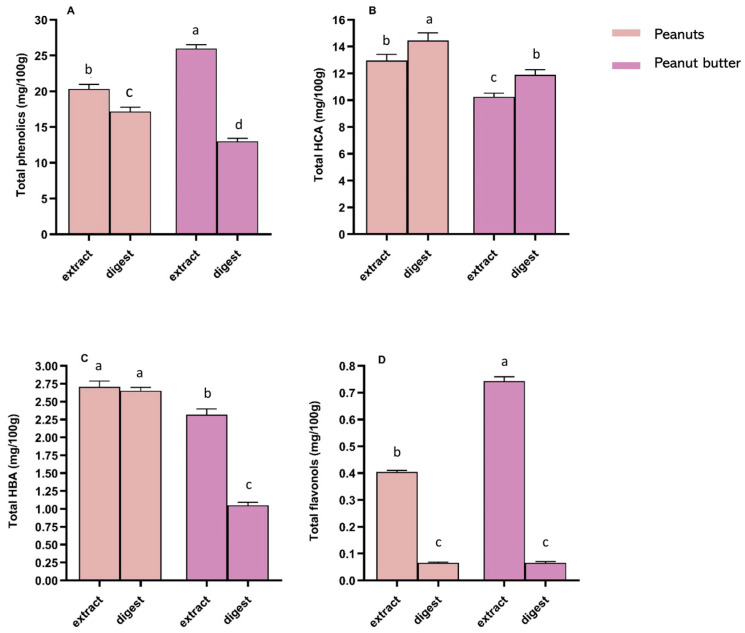
Effect of in vitro gastro-intestinal digestion on changes in total phenolic compounds (**A**), total hydroxycinnamic acids (**B**), total hydroxybenzoic acids (**C**), and total flavonols (**D**) in peanuts and peanut butter. “Extract” refers to phenolic compound extracts from peanuts and peanut butter, whereas “digest” refers to gastro-intestinally digested peanuts and peanut butter. Dusty pink bars indicate peanut samples, while purple bars indicate peanut butter samples. HBA: hydroxybenzoic acids; HCA: hydroxycinnamic acids. Different letters indicate statistically significant differences (*p* < 0.05).

A total of 55 phenolic compounds were detected and measured in peanuts and peanut butter after undergoing chemical extraction (Table 2). From a quantitative point of view, peanut butter contained 1.27-fold more total phenolic compounds (25.981 ± 0.519 mg/100 g) than peanuts (20.382 ± 0.624 mg/100 g) (Table 2 and Figure 2A). The phenolic profile differed markedly between the two matrices (Table 2 and Figure 2). In peanuts, hydroxycinnamic acids were the most representative class of phenolic compounds (63.58% of total phenolic compounds), followed by flavan-3-ols (20.75% of total phenolic compounds) and hydroxybenzoic acids (13.27% of total phenolic compounds) (Table 2 and Figure 2B,C). In contrast, flavan-3-ols were predominant in peanut butter (48.29% of total phenolic compounds), followed by hydroxycinnamic acids (39.48% of total phenolic compounds) and hydroxybenzoic acids (8.93% of total phenolic compounds) (Table 2 and Figure 2B,C).

Minor amounts of flavonols and flavones were also detected in both samples (Table 2 and Figure 2D).

In peanuts, the hydroxycinnamic acid profile was dominated by two isomers of hydroxycinnamic acids-O-pentoside, which together accounted for approximately 30% of the total phenolic compounds, along with 4-hydroxycinnamic acid (Table 2). In peanut butter, 4-hydroxycinnamic acid was the predominant hydroxycinnamic acid identified, accounting for 16.09% of the total phenolic compounds (Table 2).

Flavan-3-ols were particularly abundant in peanut butter, where two procyanidin type-A dimer isomers accounted for 36.04% of the total phenolics (Table 2). Although peanuts exhibited a similar flavan-3-ol profile, concentrations were lower (Table 2).

Vanillic acid and its isomer (hydroxy-methoxybenzoic acid) were the predominant hydroxybenzoic acids in both samples (Table 2). Four flavones, all apigenin-derivatives, were identified in both matrices. Among flavonols, isorhamnetin-3-O-rutinoside was the most abundant compound in both samples (Table 2).

The bioaccessibility index (BI) of total phenolic compounds was greater in peanuts (BI = 84.31%) than in peanut butter (BI = 50.09%). Accordingly, the absolute amount of bioaccessible phenolic compounds was significantly higher (*p* < 0.05) in peanuts (17.184 ± 0.613 mg/100 g) compared with peanut butter (13.015 ± 0.421 mg/100 g) (Table 2 and Figure 2A).

Hydroxycinnamic acids were highly stable during in vitro digestion in both samples, exhibiting BI values slightly above 100% and accounting for more than 91% of total phenolic compounds after digestion in both matrices (Table 2 and Figure 2B). This high bioaccessibility was mainly attributed to the marked stability of hydroxycinnamic acid-O-pentoside and hydroxy-methoxycinnamic acid-O-pentoside isomers, which showed BI values significantly greater than 100% (Table 2). A similar behavior was observed for the corresponding aglycones (hydroxycinnamic acids and hydroxy-methoxycinnamic acids), as well as for dimethoxy-hydroxycinnamic acid isomers, in both matrices (Table 2).

In contrast, the lower BI observed for peanut butter was primarily due to the poor stability of flavan-3-ols, which were completely degraded during in vitro digestion (Table 2).

For hydroxybenzoic acids, the BI was markedly higher in peanuts (98.08%) than in peanut butter (45.32%). This difference appears to be related to the higher proportion of hexoside-derivatives in peanut butter, which were less stable than their corresponding aglycones (Table 2 and Figure 2C). Finally, flavonols exhibited relatively low BI values in both samples, whereas flavones were no longer detected after in vitro digestion (Table 2 and Figure 2D).

### 3.3. Phenolic Compound Profile and Bioaccessibility of Pistachios and Pistachio Butter

**Figure 3:**
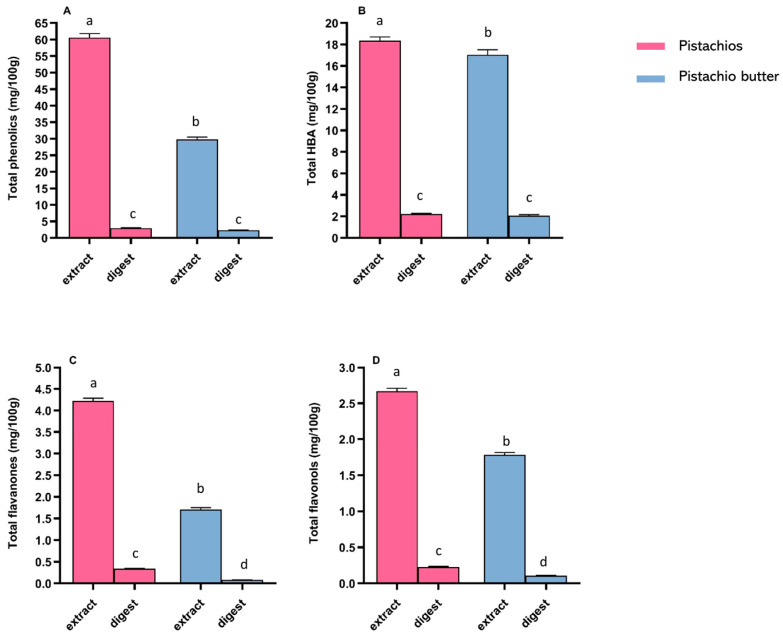
Effect of in vitro gastro-intestinal digestion on changes in total phenolic compounds (**A**), total hydroxybenzoic acids (**B**), total flavanones (**C**), and total flavonols (**D**) in pistachios and pistachio butter. “Extract” refers to phenolic compound extracts from pistachios and pistachio butter, whereas “digest” refers to gastro-intestinally digested pistachios and pistachio butter. Fuchsia bars indicate pistachio samples, while indigo bars indicate pistachio butter samples. HBA: hydroxybenzoic acids. Different letters indicate statistically significant differences (*p* < 0.05).

A total of 95 phenolic compounds were detected and quantified in pistachios and pistachio butter following chemical extraction (Table 3). From a quantitative perspective, pistachios contained 2.04-fold higher total phenolic content (60.603 ± 1.170 mg/100 g) than pistachio butter (29.758 ± 0.755 mg/100 g) (Table 3 and Figure 3A).

The phenolic profile differed markedly between the two matrices (Table 3 and Figure 3).

In pistachios, flavan-3-ols represented the predominant class of phenolic compounds, accounting for 53.08% of the total phenolic content. These were followed by hydroxybenzoic acids (30.31%), flavanones (6.97%), and flavonols (4.40%) (Table 3 and Figure 3B–D). In contrast, hydroxybenzoic acids were the dominant class in pistachio butter, representing 57.25% of the total phenolic compounds. Flavan-3-ols accounted for 24.65%, followed by flavonols (5.99%) and flavanones (5.74%) (Table 3 and Figure 3B–D). Flavones were present in appreciable amounts in both samples (4.28% in pistachios and 4.46% in pistachio butter), whereas hydroxycinnamic acids were detected only in trace amounts (Table 3).

In pistachios, procyanidin B2 was the most abundant flavan-3-ol, accounting for 23.05% of total phenolic compounds and 43.43% of total flavan-3-ols. A similar flavan-3-ols profile was observed in pistachio butter (Table 3).

In pistachio butter, hydroxybenzoic acids represented the predominant class of phenolic compounds. Among them, gallic acid-O-hexoside isomers 1 and 2 and gallic acid were the main constituents, accounting for 21.94% and 15.09% of total phenolic compounds, respectively. In contrast, pistachios exhibited a different hydroxybenzoic acid profile, with gallic acid as the most abundant compound (Table 3).

Quercetin-3-O-glucoside and two quercetin-O-hexoside isomers were the predominant flavonols in both matrices. Flavanones were mainly represented by tetrahydroxyflavanone-O-hexoside isomers, and the flavone profile was dominated by luteolin and its mono-hexoside derivatives (Table 3).

The BI of total phenolic compounds was low in both matrices but higher in pistachio butter (7.81%) than in pistachios (4.87%) (Table 3 and Figure 3A). Nevertheless, pistachios yielded a significantly greater (*p* < 0.05) absolute amount of bioaccessible phenolics after in vitro digestion (2.953 ± 0.084 mg/100 g and 2.323 ± 0.107 mg/100 g, respectively).

Flavan-3-ols, the predominant class of phenolic compounds in pistachios, showed limited stability during in vitro digestion, resulting in a substantial reduction in their relative contribution after in vitro digestion in both matrices (Table 3). The BI of hydroxybenzoic acids was similar (~12%), with gallic acid being the predominant compound after digestion, whereas gallic acid-hexoside-derivatives underwent marked degradation (Table 3 and Figure 3B). All other phenolic classes also experienced substantial degradation during digestion (Table 3 and Figure 3C,D).

### 3.4. Total Phenolic Compound and Antioxidant Activity of Nuts and Their Corresponding Butters Before and After In Vitro Digestion

**Figure 4:**
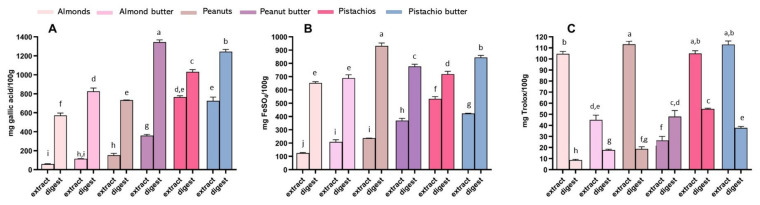
Effect of in vitro gastro-intestinal digestion on the changes in total phenolic compounds and antioxidant activity in nuts and their corresponding butters. “Extract” refers to phenolic compound extracts from nuts and butters, whereas “digest” refers to gastro-intestinally digested nuts and butters. Light pink bars indicate almond samples, lilac bars indicate almond butter samples, dusty pink bars indicate peanut samples, purple bars indicate peanut butter samples, fuchsia bars indicate pistachio samples, and indigo bars indicate pistachio butter samples. (**A**) Total phenolic compounds determined by the Folin–Ciocalteau assay. (**B**) Antioxidant activity determined by the ABTS assay. (**C**) Antioxidant activity determined by the FRAP assay. Different letters indicate statistically significant differences (*p* < 0.05).

The total phenolic content determined by the Folin–Ciocalteu assay followed the same trend as that obtained by mass spectrometry. Among the nuts, pistachio exhibited the highest total phenolic content, followed by peanuts and almonds (Figure 4A). Similarly, almond and peanut butters contained higher total phenolic levels than the corresponding nuts, whereas no significant difference (*p* > 0.05) was observed between pistachio and pistachio butter. In vitro digestion resulted in a marked increase in total phenolic content for all samples, with the highest values observed for peanut and pistachio butters (Figure 4A).

Antioxidant activity determined by the ABTS assay showed a trend consistent with total phenolic content, with pistachios and pistachio butter exhibiting the highest antioxidant capacity. Furthermore, a similar increase in antioxidant activity was observed after in vitro digestion for all samples (Figure 4B).

Before digestion, antioxidant activity assessed by the FRAP assay followed the same pattern as total phenolic content and ABTS results. In contrast, after in vitro digestion, a substantial decrease in FRAP values was observed across all samples (Figure 4C).

## 4. Discussion

The present study provides a comprehensive comparison of the phenolic profiles and in vitro bioaccessibility of almonds, peanuts, and pistachios and their corresponding butters. Distinct phenolic profiles were observed among the three nut types. Almonds were characterized by a predominance of hydroxybenzoic acids and flavonols, particularly isorhamnetin derivatives, consistent with previous reports describing almonds as a rich source of flavonol glycosides [27,28,29]. The phenolic acid profile was characterized for the presence of di-hexoside-derivatives of hydroxybenzoic and hydroxycinnamic acids, firstly identified in almond in the present study. Peanuts, in contrast, were dominated by hydroxycinnamic acids, especially pentoside-derivatives, with substantial contributions from flavan-3-ols, particularly procyanidin-type A dimers, in peanut butter. Previous studies identified hydroxycinnamic acids as the main class of phenolic compounds in peanuts, whereas flavan-3-ols were present in higher concentrations in peanut butter [1,30]. Pistachios exhibited the most distinctive profile, with flavan-3-ols, particularly procyanidins, representing more than half of the total phenolics in the nut, whereas hydroxybenzoic acids (especially gallic acid and its derivatives) dominated the phenolic profile of pistachio butter. Previous studies identified hydroxybenzoic acids and flavan-3-ols as the major phenolic compounds in pistachios [18,31,32]. Almond and peanut butters exhibited higher total phenolic concentrations than their corresponding nuts, whereas pistachios showed the opposite trend, with significantly higher levels in the whole nut compared to pistachio butter. These differences likely reflect a combination of factors, including structural disruption of the food matrix and potential losses during processing. Grinding and crushing may enhance the extractability of bound phenolics, explaining the higher concentrations measured in almond and peanut butters. A similar effect has already been hypothesized in peanuts [30]. Conversely, the substantial reduction observed in pistachio butter probably suggests degradation of labile compounds, such as flavan-3-ols, during butter production. Studies on the effect of nut butter processing on phenolic compound content are limited. In peanuts, mechanical processing has been reported to increase measurable phenolic content due to improved liberation from the matrix [30]. In general, crushing and grinding reduce particle size and disrupt cellular and subcellular structures, thereby potentially enhancing the release and extractability of phenolic compounds from nut matrices, particularly for compounds bound to cell wall polysaccharides [33]. However, the overall effect of grinding is not univocal, as processing conditions may also influence phenolic stability. For example, heat generated during milling can promote degradation of labile phenolic compounds or induce transformations such as Maillard-related reactions, which may alter phenolic composition [34].

Determination of total phenolic compounds using the Folin–Ciocalteu assay showed that pistachios contained approximately six times more total phenolics than peanuts, while almonds exhibited the lowest levels. The total phenolic contents measured in pistachios, peanuts, and almonds were consistent with values reported in previous studies [1,35,36]. In agreement with the mass spectrometry results, the total phenolic content in almond and peanut butter was higher than that of the corresponding nuts, whereas pistachio butter showed a lower phenolic content compared with pistachios. In general, the antioxidant activity determined by the ABTS assay followed the same trend as the total phenolic content. In contrast, the FRAP assay showed that almond and peanut butters exhibited lower antioxidant activity than their corresponding nuts. Correlation analysis among total phenolic content, antioxidant activity, total phenolic compounds identified by mass spectrometry, and the individual phenolic classes revealed a significant correlation between total phenolic content and ABTS values (*r* = 0.9525; *p* < 0.05), suggesting that these measurements are closely related (Supplementary Table S2). Among the phenolic subclasses, hydroxybenzoic acids and flavan-3-ols showed significant correlations with both total phenolic content and ABTS activity, indicating that these compounds may be important contributors to the antioxidant activity measured by the ABTS assay in the analyzed samples (Supplementary Table S2). In contrast, no significant correlation was observed with FRAP values. This finding suggests that the reducing capacity measured by the FRAP assay may be influenced not only by phenolic compounds but also by other sample constituents capable of reducing the ferric ions (Supplementary Table S2).

The bioaccessibility index (BI) varied substantially among nut types and phenolic classes, highlighting the importance of both molecular structure and food matrix. Peanuts showed remarkably high overall bioaccessibility (>80%), largely driven by the high stability of hydroxycinnamic acid-derivatives, many of which displayed BI values exceeding 100%. This is the case of hydroxycinnamic-pentoside-derivatives and hydroxycinnamic acids aglycones. This apparent increase after digestion may reflect hydrolysis of conjugated forms or release of bound phenolics from the matrix during gastro-intestinal conditions [37,38]. Similarly, the high BI of hydroxybenzoic acids was mainly driven by the high stability of pentoside-derivatives and methylated aglycones. The lower bioaccessibility of butter compared with nuts was almost due to the complete degradation of flavan-3-ols, which represented the main class of phenolic compounds in peanut butter. In contrast, almonds and pistachios exhibited low overall BI values (<15%), despite containing considerable total phenolic concentrations. In almonds, flavonols were the most bioaccessible class, particularly kaempferol-3-O-rutinoside and isorhamnetin-3-O-rutinoside, indicating that certain glycosylated flavonols may resist digestive degradation [15,39]. However, flavan-3-ols showed extremely poor stability (BI < 1%), confirming their well-known susceptibility to alkaline conditions and oxidative transformations during the intestinal phase [40,41]. Although total BI remained similar, almond butter delivered a greater absolute amount of bioaccessible phenolic compounds because of its higher starting concentration. Pistachios demonstrated a similar pattern, with flavan-3-ols undergoing marked degradation during digestion. Although pistachio butter showed a slightly higher BI for total phenolics compared to raw pistachios, the absolute amount of bioaccessible phenolics remained greater in the nut due to its substantially higher initial concentration. Hydroxybenzoic acids displayed moderate but consistent stability (≈12%), with gallic acid emerging as the predominant compound after digestion. The degradation of hexoside derivatives suggested that glycosidic linkages were particularly vulnerable under simulated gastro-intestinal conditions [42,43]. Therefore, relative bioaccessibility appeared to be more related to the type of nut (i.e., almond, peanut, or pistachio) than to the food matrix or processing, and mainly depended on the chemical nature of the phenolic compounds, as discussed below.

### 4.1. Bioaccessibility of Hydroxybenzoic Acids

The BI of hydroxybenzoic acids differed markedly among the nuts and their corresponding butters. In almonds, pistachios and pistachio butter, the BI for hydroxybenzoic acids was very low (<15%), whereas in almond butter the BI was higher and near to 30%. In contrast, hydroxybenzoic acids in peanuts appeared to be highly stable, particularly in the nuts, where they showed a BI of about 100%.

Hydroxy-methoxybenzoic acids (such as vanillic acid and its isomers) appeared to be more stable than the aglycones lacking a methoxy group (hydroxybenzoic and dihydroxybenzoic acids), as observed in peanuts, almonds, and their respective butters. This higher stability may be due to the presence of a methyl group esterified to a phenolic -OH group in the aromatic ring, which masks the catechol moiety and makes these compounds less susceptible to oxidative degradation [15,44]. Accordingly, the high BI of hydroxybenzoic acids in peanuts and peanut butter may be explained by the prevalence of these methylated derivatives. Moreover, peanuts also contained hydroxy-methoxybenzoic acid-O-pentosides, which appeared to be highly stable under gastro-intestinal conditions. Conversely, the corresponding hydroxybenzoic acid mono- and di-hexosides were much less stable than the pentoside-derivatives and aglycones, as observed in peanut and almond samples. The degradation of hydroxybenzoic acid mono- and di-hexosides may be attributed to deglycosylation occurring during in vitro digestion. This hypothesis is supported by the fact that, in almonds and peanuts, some hydroxybenzoic acid aglycones (especially the more stable methylated derivatives) displayed BI values above 100%. The instability of hydroxybenzoic acid mono- and di-hexosides may explain the low BI observed in almonds and almond butter. Pistachios, in contrast, were particularly rich in gallic acid and gallic acid mono-hexosides. These compounds were characterized by low BI values, and their degradation may explain the overall low BI found in pistachios and pistachio butter. The degradation of gallic acid mono-hexosides may be attributed to their deglycosylation during in vitro digestion. Previous studies have reported that gallic acid mono-hexosides were degraded under acidic conditions and underwent deglycosylation, releasing free gallic acid during the gastric phase of in vitro digestion [42,43]. Furthermore, the released gallic acid is unstable under intestinal alkaline conditions and may undergo oxidative degradation and polymerization [43,45]. Overall, the presence of a methoxy group appears to enhance the stability of hydroxybenzoic acids under gastro-intestinal conditions, as evidenced by the higher BI of hydroxy-methoxybenzoic acids (including vanillic acid) compared with hydroxybenzoic acids, di-hydroxybenzoic acids (including protocatechuic acid) and gallic acid. In contrast, the presence of a hexoside moiety reduced compound stability, as hexoside derivatives were degraded during in vitro digestion. Pentoside-derivatives, however, were considerably more stable. The higher stability of hydroxybenzoic acid-pentosides may be related to differences in the susceptibility of the glycosidic bond to acid hydrolysis and enzymatic cleavage, which are influenced by the nature of the sugar moiety.

### 4.2. Bioaccessibility of Hydroxycinnamic Acids

The BI of hydroxycinnamic acids showed marked differences among the various nuts and their corresponding butters. Very low BI values (<15%) were observed in almonds, pistachios, and their respective butters. In contrast, hydroxycinnamic acids appeared to be highly stable in peanuts and peanut butter, where BI values slightly exceeded 100%. Similar to hydroxybenzoic acids, the stability of hydroxycinnamic acids appeared to be influenced by structural features of the aglycone. In particular, the presence of at least one methyl group esterified to a phenolic –OH increased compound stability, as already observed with standard hydroxycinnamic acids [12]. This can be observed in peanuts by comparing the BI values of methoxylated derivatives with those of non-methoxylated compounds, especially caffeic acid, which contains a catechol moiety and exhibits lower stability. As observed for all samples, mono- and di-hexosides of hydroxycinnamic acids appeared to be unstable under gastro-intestinal conditions, undergoing hydrolysis and releasing the corresponding aglycones. This phenomenon was particularly evident in peanuts and peanut butter, where the decrease in glycosylated derivatives corresponded to an increase in the concentration of aglycones, which displayed BI values greater than 100%. Recently, Lopez-Rodulfo et al. [37] demonstrated that glycosylated hydroxycinnamic acids were unstable under intestinal conditions, where they underwent hydrolysis driven by pancreatin and lipase. Moreover, the presence of at least one methoxy group also appeared to enhance the stability of hydroxycinnamic acid-hexosides, as observed in almond and pistachio samples. Additionally, consistent with what was observed for hydroxybenzoic acids, pentosylated derivatives of hydroxycinnamic acids were considerably more stable than the corresponding hexosides, as shown in peanuts and peanut butter. Moreover, these compounds exhibited a BI greater than 100%, which may be attributable to their stability during digestion and to the release of matrix-bound forms. In addition, the hydrolysis of more complex derivatives, potentially not detected under the applied mass spectrometry conditions, may have contributed to the increased concentration observed after digestion. The high gastro-intestinal stability of methoxylated- and pentosylated-derivatives explains the high BI values observed in peanuts and peanut butter. In contrast, almond and pistachio samples predominantly contained hexoside-derivatives, whose instability may account for the low BI values of hydroxycinnamic acids in these samples.

### 4.3. Bioaccessibility of Flavan-3-Ols

In contrast to phenolic acids, flavan-3-ols showed extremely poor stability (BI < 1%), regardless of the sample or food matrix. In all the analyzed samples, both nuts and butters’ flavan-3-ols were mainly represented by procyanidins, predominantly in their dimeric form. Several previous studies have reported the high instability of oligomeric flavan-3-ols from different foods [41,46]. The pronounced instability of procyanidins during in vitro digestion likely reflects their high susceptibility to alkaline pH, oxidative conditions, and interactions with digestive enzymes [40,47]. In addition, their strong affinity for proteins and dietary fiber, together with their tendency to self-aggregate and precipitate, may reduce their measurable bioaccessible fraction by promoting complex formation or precipitation [46,48,49].

### 4.4. Bioaccessibility of Flavonols

The BI of flavonols displayed significant differences among the various nuts and their corresponding butters, probably reflecting the differences in the structure of the compounds and the food matrix. The lowest BI values for flavonols were observed in pistachios and pistachio butter, whereas the highest values in almonds and peanuts. Flavonols usually occur as glycosides, and the type of sugar moiety strongly influences their stability and bioaccessibility. In the majority of the samples, 3-O-rutinoside-derivatives of flavonols were more stable than the corresponding 3-O-hexoside-derivatives. Previous studies found that the presence of a rutinoside moiety in the flavonols structure enhanced stability during in vitro digestion [15,39]. The higher stability of flavonol-3-O-rutinosides compared with flavonol-3-O-glucosides during in vitro digestion may be attributed to their lower susceptibility to acid and enzymatic hydrolysis, due to the presence of a more complex disaccharide moiety as well as to a lower susceptibility to oxidation under alkaline conditions. In fact, flavonol-3-O-glucosides may undergo hydrolysis of the glycosidic bond during gastric and intestinal digestion, releasing the corresponding aglycone, and may be subject to oxidative reactions that lead to the release of the corresponding glycosylated hydroxybenzoic acids [40,50,51]. The prevalence of the 3-O-rutinoside-derivatives may explain the highest BI found in almonds and peanuts compared with pistachios, which contained mainly 3-O-hexoside-derivatives.

In the case of flavonols, a clear food matrix effect was observed since the BI values for flavonols are always lower in the butters with respect to the corresponding nuts. Grinding and homogenization during butter production may enhance the extractability of flavonols, which are more easily released during in vitro digestion, probably increasing the exposure to oxidative and alkaline degradation. A similar food matrix effect was already observed when the BI of flavonols in red-skinned onion was compared with that of a red-skinned onion phenolic extract (and therefore without the food matrix). In vitro digestion of red-skinned onion phenolic extract resulted in a stronger degradation of flavonols in the intestinal milieu compared with digested whole onion [40].

## 5. Conclusions

From a nutritional standpoint, these data indicate that total phenolic content alone is not a sufficient indicator of bioaccessibility and potential bioavailability. Although peanuts did not exhibit the highest total phenolic concentration, they showed the greatest bioaccessibility value after digestion. In contrast, pistachios, despite being the richest in phenolic compounds, demonstrated limited bioaccessibility.

Overall, these findings suggest that phenolic bioaccessibility is primarily influenced by nut type and the molecular structure of phenolic compounds, rather than by their initial concentration or the food matrix (e.g., processing into butter). The digestive stability of phenolic acids appears to depend on structural features such as methylation and glycosylation patterns. Flavonols were found to be less stable; however, the presence of a rutinoside moiety enhanced their stability compared to hexoside derivatives. Furthermore, flavan-3-ols were highly susceptible to degradation, regardless of nut type.

A major strength of this study lies in the comprehensive characterization of phenolic compounds (ranging from 55 to 95 compounds per matrix), combined with quantitative assessment of in vitro bioaccessibility.

Our study has several limitations. First, we used commercial products for which the degree of processing, technological parameters, and detailed composition were unknown. These factors may significantly influence both the phenolic profile of the products and the bioaccessibility of their phenolic compounds. Second, the findings are based on an in vitro digestion model, which provides only an estimate of bioaccessibility. Nevertheless, this approach is valuable for understanding the behavior of phenolic compounds during gastro-intestinal digestion. However, in vitro digestion models cannot fully replicate in vivo conditions, particularly in terms of absorption, metabolism, and interactions with the gut microbiota. Future studies integrating cellular uptake models or human intervention trials would be valuable to better understand the systemic bioavailability and biological relevance of these compounds.

Anyway, these findings highlight the importance of considering both chemical composition and digestive stability when evaluating the potential health benefits of nut-derived phenolic compounds.

## Figures and Tables

**Table 1:** Phenolic compound contents in almond samples were determined by mass spectrometry, reported as mg/100 g of sample. The bioaccessibility index (BI) was calculated as the percentage ratio between the concentration after in vitro intestinal digestion and that in the phenolic extract; n.d. indicates compounds not detected. Different letters within the same row denote statistically significant differences (*p* < 0.05).

Compound	Almonds					
	Almonds Extract	Digested Almonds	BI (%)	Almond Butter Extract	Digested Almond Butter	BI (%)
Hydroxybenzoic acid isomer 2	0.163 ± 0.003 ^b^	0.025 ± 0.001 ^c^	15.39	0.294 ± 0.002 ^a^	0.011 ± 0.011 ^c^	3.84
Protocatechuic acid	0.107 ± 0.002 ^b^	0.067 ± 0.005 ^c^	63.14	0.126 ± 0.007 ^a^	0.029 ± 0.002 ^d^	23.05
Vanillic acid	0.102 ± 0.002 ^c^	0.117 ± 0.011 ^c^	114.75	0.172 ± 0.004 ^b^	0.322 ± 0.005 ^a^	187.65
Hydroxy-methoxybenzoic acid-O-hexoside isomer 1	0.075 ± 0.001 ^b^	n.d.	0.00	0.120 ± 0.002 ^a^	n.d.	0.00
Hydroxy-methoxybenzoic acid-O-hexoside isomer 2	0.030 ± 0.001 ^b^	n.d.	0.00	0.057 ± 0.002 ^a^	n.d.	0.00
Hydroxy-methoxybenzoic acid-O-hexoside isomer 3	0.038 ± 0.000 ^a^	n.d.	0.00	0.032 ± 0.001 ^a^	n.d.	0.00
Hydroxy-dimethoxybenzoic acid-O-hexoside	0.008 ± 0.000 ^a^	n.d.	0.00	0.013 ± 0.000 ^a^	n.d.	0.00
Hydroxybenzoic acid-O-hexoside-hexoside	0.073 ± 0.000 ^b^	0.003 ± 0.000 ^c^	4.00	0.247 ± 0.003 ^a^	n.d.	0.00
Dihydroxybenzoic acid-O-hexoside-hexoside	0.205 ± 0.001 ^b^	0.002 ± 0.004 ^c^	1.14	0.686 ± 0.004 ^a^	0.195 ± 0.005 ^b^	28.35
Hydroxy-methoxybenzoic acid-O-hexoside-hexoside isomer 1	1.241 ± 0.012 ^b^	0.046 ± 0.001 ^d^	3.75	2.292 ± 0.027 ^a^	0.461 ± 0.003 ^c^	20.10
Hydroxy-methoxybenzoic acid-O-hexoside-hexoside isomer 2	0.312 ± 0.000 ^a^	0.066 ± 0.001 ^d^	21.09	0.107 ± 0.002 ^c^	0.170 ± 0.010 ^b^	158.45
Hydroxy-methoxybenzoic acid-O-hexoside-hexoside-hexoside	0.020 ± 0.000 ^b^	n.d.	0.00	0.079 ± 0.004 ^a^	n.d.	0.00
**Total hydroxybenzoic acids**	**2.374 ± 0.025 ^b^**	**0.327 ± 0.023 ^d^**	**13.76**	**4.223 ± 0.058 ^a^**	**1.187 ± 0.026 ^c^**	**28.10**
3-Hydroxycinnamic acid	0.002 ± 0.000 ^c^	0.008 ± 0.001 ^b^	343.62	0.013 ± 0.000 ^a^	0.007 ± 0.001 ^b^	55.91
Ferulic acid-O-hexoside isomer 1	0.046 ± 0.000 ^b^	n.d.	0.00	0.061 ± 0.000 ^a^	n.d.	0.00
Ferulic acid-O-hexoside isomer 2	0.008 ± 0.000 ^b^	0.025 ± 0.001 ^a^	0.00	0.023 ± 0.003 ^a^	n.d.	0.00
Coumaric acid-O-hexoside-hexoside isomer 1	0.041 ± 0.005 ^b^	n.d.	0.00	0.068 ± 0.001 ^a^	n.d.	0.00
Coumaric acid-O-hexoside-hexoside isomer 2	0.006 ± 0.000 ^b^	n.d.	0.00	0.023 ± 0.002 ^a^	n.d.	0.00
Caffeic acid-O-hexoside-hexoside	0.006 ± 0.000 ^b^	n.d.	0.00	0.020 ± 0.000 ^a^	n.d.	0.00
Ferulic acid-O-hexoside-hexoside isomer 1	0.889 ± 0.008 ^b^	0.025 ± 0.004 ^d^	2.79	1.265 ± 0.015 ^a^	0.060 ± 0.003 ^c^	4.72
Ferulic acid-O-hexoside-hexoside isomer 2	0.069 ± 0.001 ^b^	0.022 ± 0.001 ^c^	31.55	0.235 ± 0.011 ^a^	0.018 ± 0.001 ^c^	7.48
Dimethoxy-hydroxycinnamic acid -O-hexoside-hexoside isomer 1	0.131 ± 0.000 ^b^	0.012 ± 0.002 ^c^	9.26	0.309 ± 0.006 ^a^	0.012 ± 0.001 ^c^	3.88
Dimethoxy-hydroxycinnamic acid -O-hexoside-hexoside isomer 2	0.011 ± 0.000 ^b^	0.008 ± 0.000 ^b^	73.07	0.045 ± 0.004 ^a^	0.005 ± 0.001 ^b^	11.26
**Total hydroxycinnamic acids**	**1.209 ± 0.016 ^b^**	**0.074 ± 0.008 ^c^**	**6.13**	**2.062 ± 0.041 ^a^**	**0.102 ± 0.006 ^c^**	**4.92**
Epicatechin	0.093 ± 0.001 ^b^	0.004 ± 0.000 ^c^	3.77	0.331 ± 0.001 ^a^	0.001 ± 0.000 ^c^	0.16
Catechin	0.067 ± 0.000 ^b^	0.002 ± 0.000 ^c^	3.22	0.390 ± 0.001 ^a^	0.001 ± 0.000 ^c^	0.18
(Epi)catechin-O-hexoside isomer 1	0.083 ± 0.001 ^a^	0.002 ± 0.000 ^c^	2.84	0.067 ± 0.002 ^b^	0.001 ± 0.000 ^c^	1.76
(Epi)catechin-O-hexoside isomer 2	0.183 ± 0.001 ^a^	n.d.	0.11	0.164 ± 0.000 ^a^	n.d.	0.13
(Epi)catechin-O-hexoside isomer 3	0.113 ± 0.001 ^a^	0.003 ± 0.001 ^c^	2.72	0.059 ± 0.002 ^b^	0.004 ± 0.000 ^c^	7.24
(Epi)catechin-O-hexoside isomer 4	0.014 ± 0.001 ^a^	n.d.	1.74	0.021 ± 0.001 ^a^	0.001 ± 0.000 ^b^	2.43
(Epi)catechin-O-hexoside isomer 5	0.017 ± 0.001 ^b^	n.d.	0.70	0.033 ± 0.001 ^a^	n.d.	0.41
(Epi)catechin-O-hexoside isomer 6	0.030 ± 0.002 ^a^	n.d.	0.45	0.030 ± 0.001 ^a^	0.001 ± 0.000 ^b^	2.17
Procyanidin B1	0.295 ± 0.002 ^b^	n.d.	0.00	0.365 ± 0.008 ^a^	n.d.	0.00
(Epi)catechin-O-hexoside-hexoside	0.302 ± 0.002 ^a^	n.d.	0.00	0.219 ± 0.002 ^b^	n.d.	0.00
**Total flavan-3-ols**	**1.197 ± 0.011 ^b^**	**0.012 ± 0.001 ^c^**	**0.98**	**1.679 ± 0.019 ^a^**	**0.008 ± 0.001 ^c^**	**0.49**
Naringenin	0.050 ± 0.001 ^b^	n.d.	0.00	0.229 ± 0.001 ^a^	0.004 ± 0.001 ^c^	1.57
Tetra-hydroxyflavanone isomer 1	0.017 ± 0.000 ^b^	0.002 ± 0.000 ^c^	13.60	0.141 ± 0.000 ^a^	0.009 ± 0.001 ^c^	6.51
Tetra-hydroxyflavanone isomer 2	0.004 ± 0.000 ^b^	0.001 ± 0.000 ^b^	23.24	0.065 ± 0.001 ^a^	0.001 ± 0.001 ^b^	1.37
Naringenin-O-hexoside	0.041 ± 0.000 ^b^	n.d.	0.00	0.476 ± 0.004 ^a^	0.030 ± 0.004 ^b^	6.32
Tetra-hydroxyflavanone-O-hexoside isomer 1	0.008 ± 0.000 ^b^	0.005 ± 0.000 ^b^	59.68	0.200 ± 0.000 ^a^	0.003 ± 0.000 ^b^	1.63
Tetra-hydroxyflavanone-O-hexoside isomer 2	0.068 ± 0.001 ^b^	n.d.	0.00	0.126 ± 0.001 ^a^	0.011 ± 0.001 ^c^	8.64
Penta-hydroxyflavanone-hexoside isomer 1	0.056 ± 0.002 ^a^	n.d.	0.00	0.034 ± 0.000 ^b^	n.d.	0.00
Penta-hydroxyflavanone-hexoside isomer 2	0.076 ± 0.001 ^a^	n.d.	0.00	0.041 ± 0.000 ^b^	n.d.	0.00
Naringenin-O-hexoside-O-hexoside	0.006 ± 0.000 ^a^	n.d.	0.00	0.013 ± 0.001 ^a^	n.d.	0.00
Tetra-hydroxyflavanone-O-hexoside-hexoside isomer 1	0.017 ± 0.000 ^a^	n.d.	0.00	0.018 ± 0.000 ^a^	n.d.	0.00
Tetra-hydroxyflavanone-O-hexoside-hexoside isomer 2	0.137 ± 0.002 ^a^	n.d.	0.00	0.105 ± 0.001 ^b^	n.d.	0.00
**Total flavanones**	**0.480 ± 0.009 ^b^**	**0.008 ± 0.00 ^d^**	**1.63**	**1.448 ± 0.009 ^a^**	**0.058 ± 0.007 ^c^**	**4.00**
Kaempferol	0.005 ± 0.000 ^b^	n.d.	0.00	0.073 ± 0.000 ^a^	0.008 ± 0.005 ^b^	10.36
Quercetin	0.002 ± 0.000 ^b^	n.d.	0.00	0.028 ± 0.000 ^a^	0.001 ± 0.000 ^b^	2.90
Dihydroquercetin	0.005 ± 0.000 ^b^	0.007 ± 0.000 ^b^	135.87	0.054 ± 0.000 ^a^	n.d.	0.00
Isorhamnetin	0.013 ± 0.000 ^b^	0.005 ± 0.002 ^b^	37.27	0.072 ± 0.002 ^a^	0.008 ± 0.005 ^b^	10.89
Kaempferol-3-O-hexoside	0.048 ± 0.000 ^b^	0.005 ± 0.000 ^c^	10.54	0.117 ± 0.003 ^a^	n.d.	0.00
Quercetin-3-O-glucoside	0.010 ± 0.000 ^b^	0.001 ± 0.000 ^c^	7.65	0.034 ± 0.000 ^a^	n.d.	0.00
Quercetin-3-O-galactoside	0.009 ± 0.000 ^b^	n.d.	0.00	0.027 ± 0.001 ^a^	n.d.	0.00
Isorhamnetin-3-O-hexoside	0.321 ± 0.002 ^a^	0.040 ± 0.002 ^c^	12.50	0.295 ± 0.003 ^b^	0.027 ± 0.001 ^d^	9.24
Kaempferol-3-O-rutinoside	0.267 ± 0.001 ^b^	0.120 ± 0.002 ^d^	44.98	1.078 ± 0.009 ^a^	0.161 ± 0.004 ^c^	14.90
Kaempferol-O-hexoside-hexoside	0.011 ± 0.000 ^a^	n.d.	0.00	n.d.	n.d.	0.00
Quercetin-3-O-rutinoside	0.015 ± 0.000 ^b^	0.004 ± 0.000 ^c^	28.96	0.034 ± 0.000 ^a^	0.004 ± 0.000 ^c^	13.25
Isorhamnetin-3-O-rutinoside	0.923 ± 0.007 ^b^	0.322 ± 0.018 ^c^	34.88	1.616 ± 0.015 ^a^	0.187 ± 0.012 ^d^	11.56
**Total flavonols**	**1.628 ± 0.012 ^b^**	**0.504 ± 0.025 ^c^**	**30.96**	**3.488 ± 0.033 ^a^**	**0.395 ± 0.028 ^d^**	**11.33**
**Total phenolics MS**	**6.888 ± 0.073 ^b^**	**0.924 ± 0.057 ^d^**	**13.42**	**12.901 ± 0.160 ^a^**	**1.750 ± 0.068 ^c^**	**13.56**

**Table 2:** Phenolic compound contents in peanut samples were determined by mass spectrometry, reported as mg/100 g of sample. The bioaccessibility index (BI) was calculated as the percentage ratio between the concentration after in vitro intestinal digestion and that in the phenolic extract; n.d. indicates compounds not detected. Different letters within the same row denote statistically significant differences (*p* < 0.05).

Compound	Peanuts					
	Peanuts Extract	Digested Peanuts	BI (%)	Peanut Butter Extract	Digested Peanut Butter	BI (%)
Hydroxybenzoic acid isomer 1	0.013 ± 0.001 ^b^	0.005 ± 0.001 ^c^	41.61	0.101 ± 0.000 ^a^	0.002 ± 0.000 ^c^	2.47
Hydroxybenzoic acid isomer 3	0.019 ± 0.000 ^b^	0.006 ± 0.000 ^c^	28.89	0.044 ± 0.001 ^a^	0.005 ± 0.000 ^c^	10.28
Hydroxybenzoic acid isomer 4	0.111 ± 0.003 ^b^	0.010 ± 0.000 ^d^	9.16	0.270 ± 0.002 ^a^	0.024 ± 0.00 ^c^	8.82
Protocatechuic acid	0.004 ± 0.000 ^c^	0.003 ± 0.000 ^c^	71.45	0.032 ± 0.002 ^a^	0.011 ± 0.001 ^b^	34.71
Vanillic acid	0.247 ± 0.008 ^c^	0.657 ± 0.006 ^a^	265.45	0.481 ± 0.012 ^b^	0.280 ± 0.012 ^c^	58.20
Hydroxy-methoxybenzoic acid isomer 2	1.326 ± 0.049 ^a^	1.110 ± 0.014 ^b^	83.68	0.202 ± 0.042 ^c^	0.201 ± 0.007 ^c^	99.09
Hydroxy-methoxybenzoic acid-malic acid	0.138 ± 0.001 ^a^	0.150 ± 0.007 ^a^	108.38	0.021 ± 0.000 ^b^	0.030 ± 0.001 ^b^	144.99
Dihydroxybenzoic acid-O-pentoside	0.040 ± 0.000 ^c^	0.024 ± 0.001 ^d^	60.13	0.121 ± 0.001 ^a^	0.061 ± 0.004 ^b^	50.55
Hydroxy-methoxybenzoic acid-O-pentoside isomer 1	n.d.	0.105 ± 0.004 ^a^	NF	n.d.	0.074 ± 0.003 ^b^	NF
Hydroxy-methoxybenzoic acid-O-pentoside isomer 2	0.273 ± 0.007 ^b^	0.549 ± 0.010 ^a^	200.98	0.044 ± 0.002 ^c^	0.235 ± 0.008 ^b^	523.37
Hydroxybenzoic acid-O-hexoside isomer 1	0.031 ± 0.000 ^b^	n.d.	0.00	0.048 ± 0.000 ^a^	0.018 ± 0.001 ^c^	37.09
Hydroxybenzoic acid-O-hexoside isomer 2	0.044 ± 0.002 ^b^	n.d.	0.00	0.071 ± 0.002 ^a^	0.027 ± 0.002 ^c^	8.64
Dihydroxybenzoic acid-O-hexoside isomer 1	0.066 ± 0.001 ^b^	0.030 ± 0.001 ^c^	45.71	0.190 ±0.002 ^a^	0.064 ±0.003 ^b^	33.95
Hydroxy-methoxybenzoic acid-O-hexoside isomer 1	0.149 ± 0.003 ^b^	n.d.	0.00	0.303 ± 0.002 ^a^	0.015 ± 0.001 ^c^	4.89
Gallic acid-O-hexoside isomer 1	0.065 ± 0.001 ^b^	n.d.	0.00	0.147 ± 0.000 ^a^	n.d.	0.00
Hydroxybenzoic acid-O-acetylhexoside	0.044 ± 0.001 ^a^	n.d.	0.00	0.025 ± 0.001 ^b^	n.d.	0.00
Hydroxy-dimethoxybenzoic acid-O-hexoside	0.021 ± 0.001 ^a^	0.001 ± 0.000 ^b^	4.68	0.023 ± 0.000 ^a^	0.004 ± 0.000 ^b^	17.63
Hydroxy-methoxybenzoic acid-O-acetylhexoside isomer	0.021 ± 0.001 ^a^	0.001 ± 0.000 ^b^	4.68	0.023 ± 0.000 ^a^	0.004 ± 0.000 ^b^	17.63
Gallic acid-O-acetylhexoside	0.060 ± 0.004 ^a^	n.d.	0.00	0.037 ± 0.001 ^b^	n.d.	0.00
Hydroxy-methoxybenzoic acid-O-hexoside-pentoside	0.027 ± 0.001 ^b^	0.004 ± 0.000 ^c^	16.40	0.063 ± 0.000 ^a^	n.d.	0.00
**Total hydroxybenzoic acids**	**2.706 ± 0.082 ^a^**	**2.654 ± 0.045 ^a^**	**98.08**	**2.319 ± 0.079 ^b^**	**1.051 ± 0.043 ^c^**	**45.32**
Hydroxycinnamic acid isomer 1	0.592 ± 0.044 ^b^	0.705 ± 0.012 ^a^	119.05	0.204 ± 0.006 ^c^	0.706 ± 0.034 ^a^	345.51
4-Hydroxycinnamic acid	2.806 ± 0.015 ^b^	0.599 ± 0.010 ^c^	21.37	4.181 ± 0.050 ^a^	0.312 ± 0.012 ^d^	7.45
3-Hydroxycinnamic acid	0.352 ± 0.031 ^c^	0.450 ± 0.010 ^b^	128.03	0.245 ± 0.011 ^d^	0.501 ± 0.010 ^a^	240.06
Caffeic acid	0.072 ± 0.001 ^a^	0.039 ± 0.001 ^b^	53.70	0.061 ± 0.002 ^a^	0.026 ± 0.003 ^b^	43.31
Hydroxy-methoxycinnamic acid isomer 1	0.093 ± 0.002 ^a^	0.080 ± 0.004 ^b^	86.15	0.053 ± 0.005 ^c^	0.022 ± 0.001 ^d^	41.61
Hydroxy-methoxycinnamic acid isomer 2	0.070 ± 0.003 ^b^	0.224 ± 0.007 ^a^	319.49	0.026 ± 0.001 ^c^	0.205 ± 0.010 ^a^	786.96
Ferulic acid	0.076 ± 0.002 ^c^	0.161 ± 0.007 ^a^	211.66	0.078 ± 0.000 ^c^	0.113 ± 0.004 ^b^	144.79
Isoferulic acid	0.094 ± 0.003 ^a^	0.040 ± 0.026 ^b^	42.32	0.032 ± 0.001 ^b^	0.023 ± 0.001 ^b^	72.02
Hydroxy-methoxycinnamic acid isomer 3	0.045 ± 0.001 ^a^	0.025 ± 0.021 ^b^	54.87	0.011 ± 0.001 ^c^	n.d.	0.00
Dimethoxy-hydroxycinnamic acid isomer 1	0.036 ± 0.001 ^c^	0.148 ± 0.001 ^a^	408.37	0.038 ± 0.001 ^c^	0.123 ± 0.003 ^b^	319.99
Dimethoxy-hydroxycinnamic acid isomer 2	0.055 ± 0.002 ^c^	0.093 ± 0.005 ^a^	170.00	0.077 ± 0.002 ^b^	0.114 ± 0.004 ^a^	147.41
Dimethoxy-hydroxycinnamic acid isomer 3	0.043 ± 0.003 ^a^	0.041 ± 0.001 ^a^	95.91	0.024 ± 0.004 ^b^	0.016 ± 0.002 ^b^	67.77
Coumaroylmalic acid	0.293 ± 0.002 ^a^	0.212 ± 0.011 ^b^	72.32	0.208 ± 0.011 ^b^	0.139 ± 0.005 ^c^	66.80
Coumaric acid-O-pentoside isomer 1	0.287 ± 0.008 ^c^	1.103 ± 0.089 ^a^	383.87	0.206 ± 0.050 ^d^	0.678 ± 0.042 ^b^	329.85
Coumaric acid-O-pentoside isomer 2	3.870 ± 0.189 ^c^	4.785 ± 0.110 ^b^	123.66	2.286 ± 0.077 ^d^	5.466 ± 0.106 ^a^	239.08
Coumaric acid-O-pentoside isomer 3	2.240 ± 0.103 ^b^	2.542 ± 0.031 ^a^	113.45	0.698 ± 0.027 ^d^	1.415 ± 0.032 ^c^	202.66
Coumaric acid-O-rhamnoside isomer 1	0.045 ± 0.001 ^a^	n.d.	0.00	0.014 ± 0.001 ^b^	n.d.	0.00
Coumaric acid-O-rhamnoside isomer 2	0.072 ± 0.003 ^a^	n.d.	0.00	0.027 ± 0.000 ^b^	n.d.	0.00
Ferulic acid-O-pentoside isomer 1	0.076 ± 0.009 ^c^	0.727 ± 0.054 ^a^	956.78	0.064 ± 0.008 ^c^	0.190 ± 0.012 ^b^	296.71
Ferulic acid-O-pentoside isomer 2	0.697 ± 0.022 ^b^	1.425 ± 0.111 ^a^	204.48	0.266 ± 0.005 ^c^	1.273 ± 0.076 ^a^	478.50
Ferulic acid-O-pentoside isomer 3	0.366 ± 0.005 ^c^	0.742 ± 0.036 ^a^	202.75	0.083 ± 0.004 ^d^	0.447 ± 0.009 ^b^	538.35
Coumaric acid-O-hexoside isomer 1	0.091 ± 0.000 ^b^	n.d.	0.00	0.239 ± 0.003 ^a^	n.d.	0.00
Coumaric acid-O-hexoside isomer 2	0.005 ± 0.000 ^b^	0.032 ± 0.002 ^a^	645.76	0.040 ± 0.002 ^a^	n.d.	0.00
Caffeic acid-O-hexoside	0.020 ± 0.001 ^b^	n.d.	0.00	0.052 ± 0.001 ^a^	n.d.	0.00
Ferulic acid-O-hexoside isomer 1	0.031 ± 0.001 ^b^	n.d.	0.00	0.108 ± 0.003 ^a^	n.d.	0.00
Dimethoxy-hydroxycinnamic acid-O-pentoside	0.012 ± 0.001 ^b^	n.d.	0.00	0.040 ± 0.004 ^a^	0.002 ± 0.000 ^c^	5.15
Dimethoxy-hydroxycinnamic acid-O-hexoside isomer 1	0.061 ± 0.001 ^b^	n.d.	0.00	0.202 ± 0.001 ^a^	n.d.	0.00
Dimethoxy-hydroxycinnamic acid-O-hexoside isomer 2	0.007 ± 0.000 ^b^	n.d.	0.00	0.033 ± 0.004 ^a^	n.d.	0.00
Coumaroylmalic acid-O-hexoside	0.057 ± 0.002 ^a^	0.030 ± 0.000 ^b^	51.99	0.032 ± 0.000 ^b^	n.d.	0.00
Coumaric acid-O-hexoside-pentoside isomer 1	0.067 ± 0.000 ^c^	0.188 ± 0.012 ^a^	278.94	0.036 ± 0.000 ^d^	0.103 ± 0.007 ^b^	282.31
Coumaric acid-O-hexoside-pentoside isomer 2	0.092 ± 0.002 ^a^	0.060 ± 0.007 ^b^	65.33	0.033 ± 0.001 ^c^	0.023 ± 0.002 ^d^	73.32
Coumaric acid-O-hexoside-hexoside isomer 1	0.048 ± 0.001 ^b^	n.d.	0.00	0.129 ± 0.002 ^a^	n.d.	0.00
Coumaric acid-O-hexoside-hexoside isomer 2	0.187 ± 0.001 ^b^	n.d.	0.00	0.430 ± 0.002 ^a^	n.d.	0.00
**Total hydroxycinnamic acids**	**12.959 ± 0.462 ^b^**	**14.450 ± 0.566 ^a^**	**111.51**	**10.256 ± 0.275 ^c^**	**11.898 ± 0.374 ^b^**	**116.00**
Epicatechin	0.788 ± 0.012 ^a^	0.005 ± 0.000 ^c^	0.62	0.502 ± 0.002 ^b^	n.d.	0.00
Catechin	0.814 ± 0.006 ^a^	0.005 ± 0.000 ^c^	0.56	0.352 ± 0.001 ^b^	n.d.	0.00
(Epi)catechin-O-hexoside isomer 1	0.067 ± 0.002 ^a^	n.d.	0.00	0.052 ± 0.000 ^b^	n.d.	0.00
(Epi)catechin-O-hexoside isomer 2	0.078 ± 0.002 ^a^	n.d.	0.00	0.025 ± 0.001 ^b^	n.d.	0.00
(Epi)catechin-O-hexoside isomer 3	0.212 ± 0.002 ^a^	n.d.	0.00	0.051 ± 0.001 ^b^	n.d.	0.00
(Epi)catechin-O-hexoside isomer 4	0.635 ± 0.013 ^a^	n.d.	0.00	0.315 ± 0.010 ^b^	n.d.	0.00
(Epi)catechin-O-hexoside isomer 5	0.105 ± 0.003 ^a^	n.d.	0.00	0.009 ± 0.000 ^b^	n.d.	0.00
(Epi)catechin-O-hexoside isomer 6	0.047 ± 0.001 ^a^	0.006 ± 0.000 ^c^	12.82	0.012 ± 0.000 ^b^	n.d.	0.00
(Epi)catechin-O-acetylhexoside	0.078 ± 0.001 ^a^	n.d.	0.00	0.024 ± 0.001 ^b^	n.d.	0.00
Procyanidin-type A dimer isomer 1	0.803 ± 0.003 ^b^	n.d.	0.00	7.155 ± 0.050 ^a^	n.d.	0.00
Procyanidin-type A dimer isomer 2	0.193 ± 0.009 ^b^	n.d.	0.00	2.208 ± 0.035 ^a^	n.d.	0.00
Procyanidin B2	0.344 ± 0.010 ^b^	n.d.	0.00	1.417 ± 0.036 ^a^	n.d.	0.00
Procyanidin B1	0.065 ± 0.009 ^b^	n.d.	0.00	0.419 ± 0.009 ^a^	n.d.	0.00
(Epi)catechin-O-hexoside-hexoside	0.010 ± 0.000 ^a^	n.d.	0.00	0.007 ± 0.000 ^a^	n.d.	0.00
**Total flavan-3-ols**	**4.229 ± 0.072 ^b^**	**0.015 ± 0.001 ^c^**	**0.36**	**12.548 ± 0.146 ^a^**	**n.d.**	**0.00**
Apigenin-O-glucoside isomer 1	0.028 ± 0.001 ^a^	n.d.	0.00	0.028 ± 0.000 ^a^	n.d.	0.00
Apigenin-7-O-glucoside	0.031 ± 0.000 ^a^	n.d.	0.00	0.030 ± 0.001 ^a^	n.d.	0.00
Apigenin-C-hexoside-C-pentoside isomer 1	0.016 ± 0.000 ^b^	n.d.	0.00	0.030 ± 0.000 ^a^	n.d.	0.00
Apigenin-C-hexoside-C-pentoside isomer 2	0.010 ± 0.001 ^b^	n.d.	0.00	0.027 ± 0.001 ^a^	n.d.	0.00
**Total flavones**	**0.085 ± 0.002 ^b^**	**n.d.**	**0.00**	**0.115 ± 0.003 ^a^**	**n.d.**	**0.00**
Isorhamnetin-3-O-hexoside	0.020 ± 0.000 ^b^	0.005 ± 0.00 ^d^	24.81	0.062 ± 0.006 ^a^	0.010 ± 0.000 ^c^	16.98
Kaempferol-3-O-acetylhexoside	0.027 ± 0.000 ^b^	n.d.	0.00	0.048 ± 0.001 ^a^	n.d.	0.00
Kaempferol-3-O-rutinoside	0.083 ± 0.001 ^b^	0.013 ± 0.000 ^c^	16.08	0.135 ± 0.003 ^a^	0.013 ± 0.000 ^c^	9.53
Quercetin-3-O-rutinoside	0.053 ± 0.001 ^a^	0.009 ± 0.000 ^c^	17.06	0.027 ± 0.001 ^b^	n.d.	0.00
Isorhamnetin-3-O-rutinoside	0.221 ± 0.003 ^b^	0.038 ± 0.001 ^c^	17.32	0.400 ± 0.003 ^a^	0.031 ± 0.002 ^c^	7.75
**Total flavonols**	**0.405 ± 0.005 ^b^**	**0.066 ± 0.002 ^c^**	**16.24**	**0.744 ± 0.016 ^a^**	**0.066 ± 0.004 ^c^**	**8.87**
**Total phenolics MS**	**20.328 ± 0.624 ^b^**	**17.184 ± 0.613 ^c^**	**84.31**	**25.981 ± 0.519 ^a^**	**13.015 ± 0.421 ^d^**	**50.09**

**Table 3:** Phenolic compound contents in pistachio samples were determined by mass spectrometry, reported as mg/100 g of sample. The bioaccessibility index (BI) was calculated as the percentage ratio between the concentration after in vitro intestinal digestion and that in the phenolic extract; n.d. indicates compounds not detected. Different letters within the same row denote statistically significant differences (*p* < 0.05).

Compound	Pistachios					
	Pistachios Extract	Digested Pistachios	BI (%)	Pistachio Butter Extract	Digested Pistachio Butter	BI (%)
Hydroxybenzoic acid isomer 1	0.160 ± 0.004 ^a^	0.005 ± 0.000 ^c^	2.85	0.130 ± 0.009 ^b^	0.001 ± 0.000 ^c^	0.98
Hydroxybenzoic acid isomer 2	0.075 ± 0.004 ^b^	0.006 ± 0.000 ^c^	8.03	0.362 ± 0.010 ^a^	0.010 ± 0.001 ^c^	2.88
Hydroxybenzoic acid isomer 3	0.062 ± 0.001 ^a^	0.007 ± 0.000 ^c^	11.36	0.041 ± 0.001 ^b^	0.003 ± 0.000 ^c^	7.62
Hydroxybenzoic acid isomer 4	n.d.	n.d.	0.00	0.028 ± 0.002 ^a^	0.002 ± 0.000 ^b^	7.06
Dihydroxybenzoic acid isomer 1	0.038 ± 0.001 ^c^	0.007 ± 0.001 ^d^	18.51	0.199 ± 0.009 ^a^	0.056 ± 0.000 ^b^	28.11
Protocatechuic acid	1.198 ± 0.020	0.258 ± 0.008	21.58	0.587 ± 0.010	0.060 ± 0.002	8.60
Hydroxy-methoxybenzoic acid isomer 1	0.460 ± 0.061 ^a^	n.d.	0.00	0.636 ± 0.074 ^a^	0.071 ± 0.006 ^b^	11.13
Gallic acid	4.323 ± 0.065 ^a^	1.286 ± 0.017 ^b^	29.75	4.490 ± 0.065 ^a^	0.830 ± 0.065 ^c^	18.48
Dihydroxybenzoic acid-O-pentoside	0.137 ± 0.002 ^b^	0.046 ± 0.001 ^d^	33.77	0.246 ± 0.006 ^a^	0.059 ± 0.001 ^c^	24.15
Hydroxybenzoic acid-O-hexoside isomer 2	0.428 ± 0.002 ^a^	0.023 ± 0.001 ^c^	5.43	0.303 ± 0.007 ^b^	0.008 ± 0.001 ^d^	2.57
Dihydroxybenzoic acid-O-hexoside isomer 1	0.167 ± 0.009 ^b^	0.002 ± 0.000 ^d^	0.99	0.241 ± 0.005 ^a^	0.012 ± 0.001 ^c^	4.93
Dihydroxybenzoic acid-O-hexoside isomer 2	0.198 ± 0.006 ^a^	0.015 ± 0.000 ^c^	7.60	0.187 ± 0.008 ^a^	0.052 ± 0.001 ^b^	27.82
Dihydroxybenzoic acid-O-hexoside isomer 3	0.321 ± 0.001 ^a^	0.056 ± 0.002 ^b^	17.30	0.022 ± 0.000 ^c^	0.006 ± 0.000 ^d^	26.06
Digallic acid	0.155 ± 0.003 ^a^	n.d.	0.00	0.096 ± 0.004 ^b^	n.d.	0.00
Galloyl-shikimic acid isomer 1	0.084 ± 0.001 ^a^	n.d.	0.00	0.056 ± 0.002 ^b^	n.d.	0.00
Galloyl-shikimic acid isomer 2	0.142 ± 0.003 ^b^	0.060 ± 0.004 ^c^	42.25	0.160 ± 0.004 ^a^	n.d.	0.00
Galloyl-shikimic acid isomer 3	0.132 ± 0.004 ^a^	0.021 ± 0.001 ^c^	16.16	0.098 ± 0.001 ^b^	n.d.	0.00
Hydroxy-methoxybenzoic acid-O-hexoside isomer 1	0.118 ± 0.001 ^a^	n.d.	0.00	0.074 ± 0.003 ^b^	0.111 ± 0.007 ^a^	149.67
Hydroxy-methoxybenzoic acid-O-hexoside isomer 2	0.070 ± 0.001 ^a^	n.d.	0.00	0.053 ± 0.003 ^a^	0.004 ± 0.000 ^b^	6.96
Hydroxy-methoxybenzoic acid-O-hexoside isomer 3	0.220 ± 0.002 ^a^	n.d.	0.00	0.223 ± 0.006 ^a^	0.004 ± 0.001 ^b^	1.84
Gallic acid-O-hexoside isomer 1	2.423 ± 0.020 ^a^	0.119 ± 0.007 ^c^	4.91	1.095 ± 0.036 ^b^	0.077 ± 0.002 ^d^	7.06
Gallic acid-O-hexoside isomer 2	3.004 ± 0.036 ^b^	0.297 ± 0.007 ^d^	9.89	6.528 ± 0.161 ^a^	0.624 ± 0.006 ^c^	9.56
Syringic acid-O-hexoside	0.071 ± 0.001 ^b^	0.005 ± 0.000 ^c^	7.66	0.098 ± 0.001 ^a^	0.004 ± 0.001 ^c^	4.10
Hydroxybenzoic acid-O-hexoside-pentoside	0.224 ± 0.002 ^a^	n.d.	0.00	0.015 ± 0.001 ^b^	n.d.	0.00
Hydroxy-methoxybenzoic acid-O-hexoside-pentoside	0.060 ± 0.002 ^a^	0.005 ± 0.000 ^c^	8.23	0.079 ± 0.000 ^a^	0.016 ± 0.000 ^b^	20.48
Digallic acid-O-hexoside	0.079 ± 0.001 ^a^	n.d.	0.00	0.031 ± 0.001 ^b^	n.d.	0.00
Gallic acid-O-hexoside-O-hexoside isomer 1	0.332 ± 0.004 ^b^	n.d.	0.00	0.586 ± 0.021 ^a^	n.d.	0.00
Gallic acid-O-hexoside-O-hexoside isomer 2	0.039 ± 0.002 ^c^	n.d.	0.00	0.101 ± 0.005 ^a^	0.063 ± 0.002 ^b^	62.65
Trigallic acid-O-hexoside	0.103 ± 0.002 ^a^	n.d.	0.00	0.088 ± 0.002 ^b^	n.d.	0.00
Tetragallic acid-O-hexoside	0.979 ± 0.024 ^a^	n.d.	0.00	0.236 ± 0.011 ^b^	n.d.	0.00
Pentagallic acid-O-hexoside	2.565 ± 0.055	n.d.	0.00	n.d.	n.d.	0.00
Ellagic acid	n.d.	0.011 ± 0.001 ^a^	NF	n.d.	0.002 ± 0.000 ^b^	NF
**Total hydroxybenzoic acids**	**18.366 ± 0.338 ^a^**	**2.230 ± 0.051 ^c^**	**12.14**	**17.038 ± 0.467 ^b^**	**2.066 ± 0.097 ^c^**	**12.13**
Hydroxycinnamic acid isomer 1	0.053 ± 0.002 ^a^	n.d.	0.00	0.028 ± 0.001 ^b^	0.007 ± 0.000 ^c^	25.98
3-Hydroxycinnamic acid	0.045 ± 0.000 ^a^	n.d.	0.00	0.032 ± 0.003 ^a^	0.006 ± 0.000 ^b^	18.05
Hydroxy-methoxycinnamic acid isomer 1	0.087 ± 0.001 ^a^	n.d.	0.00	0.072 ± 0.001 ^b^	n.d.	0.00
Ferulic acid-O-hexoside isomer 1	0.318 ± 0.004 ^a^	n.d.	0.00	0.246 ± 0.009 ^b^	n.d.	0.00
Ferulic acid-O-hexoside isomer 2	0.021 ± 0.001 ^b^	0.021 ± 0.001 ^b^	100.00	0.149 ± 0.004 ^a^	n.d.	0.00
Dimethoxy-hydroxycinnamic acid-O-hexoside isomer 2	0.061 ± 0.000 ^a^	0.061 ± 0.000 ^a^	100.00	0.040 ± 0.000 ^b^	n.d.	0.00
**Total hydroxycinnamic acids**	**0.586 ± 0.008 ^a^**	**0.082 ± 0.001 ^b^**	**14.02**	**0.568 ± 0.017 ^a^**	**0.013 ± 0.000 ^c^**	**2.31**
Epicatechin	2.950 ± 0.054 ^a^	0.008 ± 0.000 ^c^	0.27	1.141 ± 0.005 ^b^	0.013 ± 0.000 ^c^	1.14
Catechin	2.116 ± 0.020 ^a^	0.005 ± 0.002 ^c^	0.24	0.253 ± 0.004 ^b^	0.003 ± 0.000 ^c^	1.18
Epigallocatechin	2.613 ± 0.013 ^a^	n.d.	0.00	1.557 ± 0.033 ^b^	0.008 ± 0.000 ^c^	0.53
Gallocatechin	0.147 ± 0.003 ^a^	n.d.	0.00	0.135 ± 0.005 ^a^	n.d.	0.00
Catechin-3-O-gallate	0.365 ± 0.017 ^a^	n.d.	0.00	0.107 ± 0.003 ^b^	n.d.	0.00
Epicatechin-3-O-gallate	1.571 ± 0.024 ^a^	n.d.	0.00	0.399 ± 0.007 ^b^	n.d.	0.00
(Epi)catechin-O-hexoside isomer 1	0.047 ± 0.000 ^a^	n.d.	0.00	0.027 ± 0.002 ^b^	n.d.	0.00
(Epi)catechin-O-hexoside isomer 2	0.058 ± 0.000 ^b^	0.002 ± 0.000 ^c^	3.22	0.081 ± 0.001 ^a^	n.d.	0.00
(Epi)catechin-O-hexoside isomer 3	0.163 ± 0.001 ^a^	0.005 ± 0.000 ^b^	2.83	0.006 ± 0.000 ^b^	n.d.	0.00
(Epi)catechin-O-hexoside isomer 4	0.030 ± 0.001 ^a^	n.d.	0.00	0.006 ± 0.001 ^b^	n.d.	0.00
(Epi)catechin-O-hexoside isomer 5	0.011 ± 0.000 ^a^	n.d.	0.00	0.006 ± 0.000 ^a^	n.d.	0.00
Epigallocatechin-3-O-gallate	0.294 ± 0.005 ^a^	n.d.	0.00	0.197 ± 0.004 ^b^	n.d.	0.00
Procyanidin B2	13.971 ± 0.369 ^a^	0.006 ± 0.001 ^c^	0.04	2.251 ± 0.062 ^b^	n.d.	0.00
Procyanidin B1	1.885 ± 0.025 ^a^	n.d.	0.00	0.480 ± 0.009 ^b^	n.d.	0.00
(Epi)catechin-O-hexoside-hexoside	0.038 ± 0.001 ^b^	n.d.	0.00	0.053 ± 0.001 ^a^	n.d.	0.00
Procyanidin-type B trimer isomer 1	2.399 ± 0.035 ^a^	n.d.	0.00	0.250 ± 0.002 ^b^	n.d.	0.00
Procyanidin-type B trimer isomer 2	1.163 ± 0.047 ^a^	n.d.	0.00	0.148 ± 0.010 ^b^	n.d.	0.00
Procyanidin-type B trimer isomer 3	2.348 ± 0.061 ^a^	n.d.	0.00	0.236 ± 0.010 ^b^	n.d.	0.00
**Total flavanols**	**32.169 ± 0.676 ^a^**	**0.025 ± 0.002 ^c^**	**0.08**	**7.334 ± 0.159 ^b^**	**0.024 ± 0.001 ^c^**	**0.33**
Tetra-hydroxy-flavone isomer 1	0.090 ± 0.003 ^a^	0.002 ± 0.000 ^b^	2.44	0.089 ± 0.002 ^a^	0.002 ± 0.000 ^b^	1,81
Luteolin	0.543 ± 0.014 ^a^	0.007 ± 0.003 ^c^	1.27	0.167 ± 0.002 ^b^	0.002 ± 0.000 ^a^	1.31
Apigenin-O-glucoside isomer 1	0.004 ± 0.000 ^b^	n.d.	0.00	0.027 ± 0.001 ^a^	n.d.	0.00
Apigenin-7-O-glucoside	0.053 ± 0.001 ^a^	n.d.	0.00	0.032 ± 0.002 ^b^	n.d.	0.00
Luteolin-O-hexoside isomer 1	1.142 ± 0.010 ^a^	0.019 ± 0.002 ^c^	1.62	0.359 ± 0.007 ^b^	n.d.	0.00
Luteolin-O-hexoside isomer 2	n.d.	0.001 ± 0.000 ^b^	NF	0.026 ± 0.002 ^a^	0.002 ± 0.000 ^b^	6.20
Luteolin-O-hexoside isomer 3	0.070 ± 0.002 ^a^	0.002 ± 0.000 ^b^	2.58	0.073 ± 0.003 ^a^	0.003 ± 0.000 ^b^	3.44
Luteolin-O-hexoside isomer 4	n.d.	0.018 ± 0.002 ^b^	NF	0.047 ± 0.002 ^a^	0.016 ± 0.001 ^b^	34.64
Luteolin-O-hexoside isomer 5	0.044 ± 0.001 ^b^	0.002 ± 0.000 ^c^	5.28	0.168 ± 0.010 ^a^	n.d.	0.00
Luteolina-7-O-glucoside	0.645 ± 0.009 ^a^	0.001 ± 0.000 ^c^	0.16	0.338 ± 0.004 ^b^	0.008 ± 0.000 ^c^	2.38
**Total flavones**	**2.592 ± 0.041 ^a^**	**0.052 ± 0.007 ^c^**	**2.00**	**1.326 ± 0.035 ^b^**	**0.032 ± 0.002 ^c^**	**2.42**
Naringenin	0.078 ± 0.001 ^a^	0.001 ± 0.000 ^c^	1.22	0.014 ± 0.000 ^b^	n.d.	0.00
Tetra-hydroxyflavanone isomer 1	0.442 ± 0.005 ^a^	0.010 ± 0.001 ^c^	2.23	0.154 ± 0.003 ^b^	0.004 ± 0.000 ^c^	2.32
Penta-hydroxyflavanone isomer 1	0.153 ± 0.001 ^a^	0.015 ± 0.001 ^c^	9.93	0.139 ± 0.003 ^b^	0.011 ± 0.000 ^c^	7.89
Penta-hydroxyflavanone isomer 2	0.044 ± 0.001 ^a^	0.003 ± 0.000 ^b^	5.82	0.036 ± 0.002 ^a^	0.002 ± 0.000 ^b^	4.14
Naringenin-O-hexoside isomer	0.119 ± 0.002 ^a^	n.d.	0.00	0.044 ± 0.000 ^b^	n.d.	0.00
Tetra-hydroxyflavanone-O-hexoside isomer 1	1.230 ± 0.011 ^a^	0.237 ± 0.004 ^c^	19.26	0.510 ± 0.012 ^b^	0.046 ± 0.000 ^d^	8.92
Tetra-hydroxyflavanone-O-hexoside isomer 2	0.968 ± 0.010 ^a^	0.011 ± 0.000 ^c^	0.11	0.463 ± 0.009 ^b^	0.012 ± 0.001 ^c^	2.57
Penta-hydroxyflavanone-hexoside isomer 1	0.030 ± 0.001 ^b^	n.d.	0.00	0.061 ± 0.010 ^a^	n.d.	0.00
Penta-hydroxyflavanone-hexoside isomer 2	0.470 ± 0.016 ^a^	0.053 ± 0.004 ^c^	11.21	0.287 ± 0.003 ^b^	0.008 ± 0.001 ^d^	2.68
Penta-hydroxyflavanone-hexoside isomer 3	0.687 ± 0.016 ^a^	0.011 ± 0.000 ^b^	1.54	n.d.	n.d.	0.00
**Total flavanones**	**4.222 ± 0.062 ^a^**	**0.340 ± 0.011 ^c^**	**8.04**	**1.709 ± 0.043 ^b^**	**0.081 ± 0.003 ^d^**	**4.75**
Quercetin	0.282 ± 0.003 ^a^	0.012 ± 0.006 ^c^	4.11	0.132 ± 0.002 ^b^	n.d.	0.00
Isorhamnetin	0.029 ± 0.000 ^a^	n.d.	0.00	0.030 ± 0.001 ^a^	n.d.	0.00
Quercetin-O-hexoside isomer 1	0.438 ± 0.006 ^a^	0.001 ± 0.000 ^c^	0.30	0.182 ± 0.000 ^b^	n.d.	0.00
Quercetin-3-O-glucoside	0.460 ± 0.001 ^a^	0.050 ± 0.000 ^c^	10.81	0.315 ± 0.007 ^b^	0.025 ± 0.001 ^d^	7.86
Quercetin-3-O-galactoside	0.703 ± 0.021 ^a^	0.077 ± 0.002 ^c^	10.90	0.301 ± 0.003 ^b^	0.038 ± 0.001 ^d^	12.64
Isorhamnetin-O-hexoside isomer 1	0.100 ± 0.001 ^a^	0.007 ± 0.000 ^c^	6.55	0.029 ± 0.000 ^b^	0.007 ± 0.000 ^c^	24.47
Isorhamnetin-3-O-hexoside	0.044 ± 0.000 ^b^	n.d.	0.00	0.059 ± 0.003 ^a^	n.d.	0.00
Quercetin-3-O-rutinoside	0.196 ± 0.002 ^a^	0.058 ± 0.000 ^c^	29.48	0.147 ± 0.001 ^b^	0.019 ± 0.001 ^d^	13.24
Quercetin-O-galloylhexoside isomer 1	0.113 ± 0.003 ^a^	n.d.	0.00	0.085 ± 0.002 ^b^	n.d.	0.00
Quercetin-O-galloylhexoside isomer 2	0.042 ± 0.002 ^a^	n.d.	0.00	0.027 ± 0.000 ^b^	n.d.	0.00
Isorhamnetin-3-O-rutinoside	0.055 ± 0.001 ^a^	0.008 ± 0.001 ^b^	14.27	0.052 ± 0.002 ^a^	0.007 ± 0.001 ^b^	14.21
Quercetin-O-hexoside-O-hexoside isomer 1	0.015 ± 0.000 ^b^	0.001 ± 0.001 ^c^	5.95	0.029 ± 0.001 ^a^	0.002 ± 0.000 ^c^	6.69
Quercetin-O-hexoside-O-hexoside isomer 2	0.047 ± 0.001 ^b^	0.007 ± 0.000 ^c^	14.27	0.132 ± 0.005 ^a^	0.006 ± 0.000 ^c^	4.39
Quercetin-O-hexoside-O-hexoside isomer 3	n.d.	0.001 ± 0.000 ^b^	NF	0.019 ± 0.000 ^a^	0.002 ± 0.001 ^b^	10.16
Quercetin-O-hexoside-O-hexoside isomer 4	0.070 ± 0.001 ^b^	0.003 ± 0.001 ^c^	4.53	0.128 ± 0.004 ^a^	n.d.	0.00
Quercetin-O-hexoside-O-hexoside isomer 5	0.016 ± 0.000 ^b^	0.001 ± 0.000 ^c^	8.62	0.027 ± 0.001 ^a^	n.d.	0.00
Quercetin-O-hexoside-O-hexoside isomer 6	0.011 ± 0.000 ^b^	n.d.	2.81	0.036 ± 0.001 ^a^	n.d.	0.00
Isorhamnetin-O-hexoside-O-hexoside	0.019 ± 0.000 ^a^	n.d.	0.00	0.025 ± 0.000 ^a^	n.d.	0.00
Quercetin-tri-O-hexoside	0.027 ± 0.000 ^a^	n.d.	0.00	0.030 ± 0.000 ^a^	n.d.	0.00
**Total flavonols**	**2.668 ± 0.044 ^a^**	**0.225 ± 0.011 ^c^**	**8.43**	**1.783 ± 0.034 ^b^**	**0.106 ± 0.005 ^c^**	**5.96**
**Total phenolics MS**	**60.603 ± 1.170 ^a^**	**2.953 ± 0.084 ^c^**	**4.87**	**29.785 ± 0.755 ^b^**	**2.323 ± 0.107 ^c^**	**7.81**

## Data Availability

The original contributions presented in the study are included in the article/Supplementary Materials. Further inquiries can be directed to the corresponding author.

## Supplementary Materials

The following supporting information can be downloaded at: https://www.mdpi.com/article/10.3390/foods15132302/s1, Table S1: Mass spectrometry data of identified phenolic compounds. Table S2: Correlation analysis among antioxidant activity assays and phenolic compounds profiles of the different nut samples. Data are shown as pearson correlation coefficient (denoted as *r*). Statistically significant data (*p* < 0.05) are highlighted in yellow.
