# Supplementary material for: Comparative Bioaccessibility of Phenolic Compounds from Almonds, Peanuts, Pistachios and Their Corresponding Butters
Source: Foods. 2026 Jun 27;15(13):2302. doi: 10.3390/foods15132302 (PMC13362184; doi:10.3390/foods15132302)
Supplement: Supplementary file 1 [file foods-15-02302-s001.zip › Supplementary_Table_S2.pdf]

Supplementary Table S2. Correlation analysis among antioxidant activity assays and phenolic compounds profiles of the different nut samples. Data are shown as pearson correlation coefficient (denoted as *r*). Statistically significant data ($P < 0.05$) are highlighted in yellow.

	Total phenolic compounds (Folin)	ABTS	FRAP	Total phenolic compounds (MS)	Total hydroxybenzoic acids	Total flavonols	Total hydroxycinnamic acids	Total flavan-3-ols
Total phenolic compounds (Folin)	/	0.9525	0.2724	0.8632	0.9327	0.1516	-0.3697	0.7572
ABTS	0.9525	/	0.0834	0.9352	0.8234	0.1053	-0.1761	0.8648
FRAP	0.2724	0.0834	/	0.2161	0.4619	-0.1422	-0.2391	0.0952
